# Natural Product Cordycepin (CD) Inhibition for NRP1/CD304 Expression and Possibly SARS-CoV-2 Susceptibility Prevention on Cancers

**DOI:** 10.3390/microorganisms11122953

**Published:** 2023-12-10

**Authors:** Ting Li, Na Luo, Jiewen Fu, Jiaman Du, Zhiying Liu, Qi Tan, Meiling Zheng, Jiayue He, Jingliang Cheng, Dabing Li, Junjiang Fu

**Affiliations:** 1 Key Laboratory of Epigenetics and Oncology, The Research Center for Preclinical Medicine, Southwest Medical University, Luzhou 646000, China; 20220199120034@stu.swmu.edu.cn (T.L.); 20220199120033@stu.swmu.edu.cn (N.L.); fujiewen@swmu.edu.cn (J.F.); 20190199120004@stu.swmu.edu.cn (J.D.); 20210199120022@stu.swmu.edu.cn (Z.L.); 20210199120024@stu.swmu.edu.cn (Q.T.); zhengmeiling@swmu.edu.cn (M.Z.); hejieyue@swmu.edu.cn (J.H.); jingliangc@swmu.edu.cn (J.C.); 2 School of Basic Medical Sciences, Southwest Medical University, Luzhou 646000, China

**Keywords:** the *NRP1/CD304* gene, SARS-CoV-2, cancers, cordycepin (CD), therapeutics

## Abstract

NRP1/CD304 is a typical membrane-bound co-receptor for the vascular endothelial cell growth factor (VEGF), semaphorin family members, and viral SARS-CoV-2. Cordycepin (CD) is a natural product or active gradient from traditional Chinese medicine (TCM) from *Cordyceps militaris* Link and *Ophiocordycep*s sinensis (Berk.). However, NRP1 expression regulation via CD in cancers and the potential roles and mechanisms of SARS-CoV-2 infection are not clear. In this study, online databases were analyzed, Western blotting and quantitative RT-PCR were used for NRP1 expression change via CD, molecular docking was used for NRP/CD interaction, and a syncytial formation assay was used for CD inhibition using a pseudovirus SARS-CoV-2 entry. As a result, we revealed that CD inhibits NRP1 expressed in cancer cells and prevents viral syncytial formation in 293T-hACE2 cells, implying the therapeutic potential for both anti-cancer and anti-viruses, including anti-SARS-CoV-2. We further found significant associations between NRP1 expressions and the tumor–immune response in immune lymphocytes, chemokines, receptors, immunostimulators, immune inhibitors, and major histocompatibility complexes in most cancer types, implying NRP1’s roles in both anti-cancer and anti-SARS-CoV-2 entry likely via immunotherapy. Importantly, CD also downregulated the expression of NRP1 from lymphocytes in mice and downregulated the expression of A2AR from the lung cancer cell line H1975 when treated with CD, implying the NRP1 mechanism probably through immuno-response pathways. Thus, CD may be a therapeutic component for anti-cancer and anti-viral diseases, including COVID-19, by targeting NRP1 at least.

## 1. Introduction

The NRP1 (Neuropilin 1, OMIM: 602069), CD304/VEGF165R/NRP/vascular endothelial cell growth factor 165 receptor, is a typical membrane-bound co-receptor for both members of the semaphorin family and vascular endothelial growth factor (VEGF) [1,2,3,4]. NRP1 is a cytogenetic located on the human chromosome 10p11.22. NRP1 encodes the deduced 923-amino acid protein with a molecular mass of 103,134 Da (NM_003873.7, NP_003864.5) containing an N-terminal signal sequence, a transmembrane region, an ectodomain, and a cytoplasmic domain, which is consistent to the structure of cell surface receptors [5]. These specific domains participated in different signaling pathways and versatile roles controlling survival, migration, and invasion, as well as angiogenesis and axon guidance, through binding ligands to co-receptors, including VEGF and semaphorin family members [3,4]. For example, in breast cancer cells MDA-MB-231, CRISPR-Cas9 knocking out the *NRP1* gene was reported to have a pronounced reduction in lung metastasis [6].

Cantuti-Castelvetri et al. [7] and Daly et al. [8] found that NRP1 can act as a receptor to mediate severe acute respiratory syndrome coronavirus 2 (SARS-CoV-2) invading host cells [9]. As we well know, SARS-CoV-2 caused the severe coronavirus disease 2019 (COVID-19), leading to a global pandemic since the outbreak at the end of 2019 [10,11,12]. Unlike the S-protein of SARS-CoV, the SARS-CoV-2 S-protein has a polybasic sequence domain (Arg-Arg-Ala-Arg) (the C-end rule, CendR) at the S1-S2 boundary that facilitates the cleavage via furin [13], an enzyme convertase that catalyzes the conversion of a substance to its active state. Thus, SARS-CoV-2 can easily enter the host cells with the aid of NRP1, nourishing its infectivity and promoting its tropism [14]. In addition, cells from the bronchioalveolar lavage of COVID-19 patients showed an increase in NRP1 RNA expressions in SARS-CoV-2 positive cells but not in uninfected cells [7], further enhancing SARS-CoV-2 entry. Tumor necrosis factor α (TNFα) and interleukin-1β (IL-1β) increased the expression of NRP2, an isoform of NRP1, and promoted SARS-CoV-2 proliferation and S-protein binding, thus revealing proinflammatory cytokines such as TNFα for the contribution of SARS-CoV-2 proliferation in host human cells [15]. Meanwhile, Wang et al. [16] reported that NRP1 is highly expressed in macrophages and dendritic cells (DCs) from inside myeloid lineage cells but not in CD4+ T cells, acting as an inhibitor of HIV-1 infectivity.

Gene polymorphisms within the receptors/co-receptors of SARS-CoV-2, including NRP1 (rs10080), were reported to associate with variable COVID-19 outcomes across ethnicities [17,18]. Mutating NRP1 novel interaction sites, located in the vestigial plasminogen–apple–nematode (PAN) domain, were recently reported to reduce the S-protein of SARS-CoV-2 internalization [19], although another reported that the binding affinity is almost the same after mutation at some NRP1 sites (rs141633354, rs142121081, rs145954532, rs200660300, rs200028992, rs369312020, rs370551432, rs370641686, and rs370117610) via molecular docking [20].

Nevertheless, targeting NRP1 could be a potential approach to preventing SARS-CoV-2 entry [21,22] and for developing potential anti-tumor drugs [23,24], with a peptide-based inhibition in anti-angiogenesis, anti-proliferation, and anti-migration of tumor cells [25]. In addition, NRP1 also facilitated other various viruses’ invasion and replication, such as the Epstein–Barr virus (EBV) [26], the pseudorabies virus (PRV) [27], the mouse cytomegalovirus (mCMV) [28], and the retroviruses for the human T cell lymphotropic virus-1 (HTLV-1) and HTLV-2 [29].

Small-molecule inhibitors for the S-protein in SARS-CoV-2 may bind to NRP1 [30]. In silico analysis found that interfering with SARS-CoV-2 binds to NRP1 via small molecules of natural products seem to be potential candidates as novel anti-viral agents [31,32,33,34]. Folic acid, leucovorin, and alimemazine may have the potential to prevent SARS-CoV-2 internalization by interacting with the S-protein/NRP1 complex [35,36]. Targeting NRP1 with small molecules would thus have the potential to interfere with SARS-CoV-2 invasion [37]. However, NRP1 expression in pan-cancers, its regulation, and the potential role of SARS-CoV-2-infected cancer patients are not clear. It is essential to identify novel small molecules from natural products or traditional Chinese medicine (TCM) with anti-tumor functions that can modulate the expression of host cell entry regulators for interfering with SARS-CoV-2 entry [14,30,38]. Cordycepin (CD) is an active gradient or natural product from traditional Chinese medicine (TCM) from *Cordyceps militaris* Link and *Ophiocordycep*s sinensis (Berk.). Cordycepin (CD), an adenosine derivative, processes a diverse, broad spectrum of biological/pharmacological activities, such as anti-cancer, antimetastatic, anti-viral, antiprotozoal, antimalarial, antimicrobial, insecticidal, anti-inflammatory, antioxidant, and immunomodulatory/immunoregulatory [39,40,41].

However, NRP1 expression regulation by CD in cancers, and the potential role and mechanism of SARS-CoV-2 infection are not clear [42,43]. In this study, we analyzed the NRP1 expressions and viral syncytial formation in cancer cell lines. Molecular docking was used to investigate NRP/CD interaction. The changes in the immune molecules from lymphocytes in mice when treated with CD were also conducted. Thus, CD may be a therapeutic component for SARS-CoV-2 and cancers by at least targeting NRP1.

## 2. Materials and Methods

### 2.1. Online Databases

An integrated repository portal for tumor–immune system interactions (TISIDB) was applied to perform the correlations between abundance in tumor-infiltrating lymphocytes (TILs) and NRP1 expression (http://cis.hku.hk/TISIDB/browse.php?gene=NRP1) (accessed on 1 January 2023) [44]. The sequences for the *NRP1* gene from GenBank NM_003873.7 in the National Center for Biotechnology Information (NCBI) were used to design quantitative RT-PCR primers [11]. Primer 3 (v. 0.4.0) was used to design NRP1 PCR primers and other gene primers (https://bioinfo.ut.ee/primer3-0.4.0/) (accessed on 1 January 2023) [45].

### 2.2. Antibodies and Reagents

The NRP1 antibody was purchased from the company of Santa Cruz Biotechnology (sc-5307, Dallas, TX, USA). β-actin and HSP70 antibodies served as an internal control. The CD was previously described [38] and purchased from Must Bio-Technology Co., Ltd., Chengdu, China. Fetal bovine serum (FBS) (cat.no.: A6907) was purchased from Invigentech (Irvine, CA, USA). The Roswell Park Memorial Institute (RPMI) 1640 medium (cat. no.: C3010-0500) or Dulbecco’s modified Eagle’s medium (DMEM) (cat. no.: C3113-0500) plus a 10% FBS was used for cell culture [40,46,47].

### 2.3. Cell Culture

The indicated cancer cells (H1975, BT549, PC3, and 22RV1) were used, and cultured conditions for cells have been previously described using an RPMI 1640 medium or a DMEM medium with a 10% FBS with antibiotics at 37 °C in a 5% CO_2_ [38,48]. In addition, the 293T-hACE2 cell lines were gifted from Professor Xianghui Fu [49] and cultured using the DMEM medium with a 10% FBS with antibiotics at 37 °C in a 5% CO_2_.

### 2.4. CD Treatments and Isolation of Mouse Lymphocytes

BALB/c female mice, which were purchased at Tengxin Biotechnology Co., Ltd. (Chongqing, China), were fed under a constant temperature at 22 °C, 50–60% humidity, and a light/dark cycle for 12 h according to the feeding standard. Six BALB/c female mice were selected and divided into two groups: the experimental and control groups. The mice (10 weeks old, about 24 g) were injected CD with 25 mg/kg/mouse (three mice per group) through the caudal vein and showed no abnormalities, which were observed once every 12 h. After 24 h, sodium pentobarbital (200 mg/kg body weight) was injected intraperitoneally in the experimental group and euthanized. Death presenting no heartbeat after anesthesia, dilated pupils, or cervical dislocation were confirmed as non-vital signs.

A CD (0.006 mg/μL) solution (containing 20% DMSO, 30% polyethylene glycol 400, 5% Tween 80, and 63% NaCl) with 25 mg/kg (CD/mouse) was injected into mice. After 24 h, T cells were isolated from the spleens of mice using our mouse T cell isolationprotocol (>95% purity) with the red blood cell lysate. For details, the spleens of the sacrificed mice were isolated, ground, and filtered using cell strainers (size: 100 μM, cat. no.: 15-1100; Biologix Group Ltd., Camarillo, CA, USA) under an ice bath, then collected into a 15 mL tube and centrifuged. The supernatant was lysed with an iced 1×red blood cell lysis solution (150 mM NH4Cl, 10 mM KHCO_3_, and 0.1 mM EDTA) and mixed well. After lysis for 5–8 min on ice, the reaction was terminated by a cold 1 × PBS. After centrifugation, the supernatant was mixed with ice 1 × PBS, and the debris was discarded. Then, each sample containing mouse lymphocytes was used for protein and RNA extraction, respectively.

### 2.5. Western Blotting

After treatments with indicated drugs (0, 10, 20, and 40 μM), the cells were washed and lysed with an ice-cold 1 × EBC buffer (20 mM Tris-HCl pH8.0, 125 mM NaCl, 2 mM EDTA, 0.5% NP-40, and protease inhibitors). Then, the 2× SDS (sodium dodecyl sulfate) buffer was added, and the extracted samples were boiled at 100 °C for 5 min. About 50 μg samples in each well were taken for SDS–PAGE electrophoresis, and proteins were separated into 8% or 10% gels according to the molecular weight sizes of proteins with a voltage of 100 V for 2 h. After electrophoresis, the proteins were transferred into the PVDF membranes (polyvinylidene fluoride) with a voltage of 100 V for 2 h. The 1 × TBST (Tris-buffered saline with Tween 20, 137 mM NaCl, 2.7 mM KCl, 25 mM Tris, and 0.05% Tween 20) was used to wash the membranes to remove the extra methanol three times at room temperature. Then, the membranes were blocked with 5% nonfat milk in TBST for 2 h. After being washed in 1 × TBST three times, the membranes were cut, and the proteins were incubated with the indicated NRP1 and β-actin/HSP70 antibodies in 2% nonfat milk in 1 × TBST at 4 °C all night. The next day, 1 × TBST was used to wash the membranes to remove the extra primary antibodies three times, and then, the secondary antibodies were added with 2% nonfat milk in 1 × TBST for 2 h; then, 1 × TBST was washed for three times. Finally, the bands on the membranes were measured under the image scanner (Gene Company Limited, Gbox Chemi, DRXV4/1068, Hong Kong, China) after adding the Super Signal West Femto Maximum Sensitivity Substrate (Thermo Fish Scientific, XG346245, Boston, MA, USA) and BeyoECL Plus (P0018S, Shanghai, China). All experiments were repeated three times.

### 2.6. Semi-Quantitative RT-PCR

The CD-treated cells were extracted using RNA; then, the mRNA was reversely transcribed into cDNA with reverse transcriptase. A semi-quantitative RT-PCR was performed using NRP1 RT-PCR primers and ACTB RT-PCR primers using the above cDNA as a template. The RT-PCR primers for *NRP1* were as follows: RT-NRP1-5:5′-ccacagtggaacaggtgatg-3′ and RT-NRP1-3:5′-cgtactcctctggcttctgg-3′. The product size was 416 bp. The RT-PCR primers for *A2AR* (Genbank No. NM_000675.6) were as follows: RT-A2AR-L: 5′-tcaacagcaacctgcagaac-3′ and RT-A2AR-R: 5′-tccaacctagcatgggagtc-3′. The product size was 333 bp. *ACTB* was set up as an internal control with 510 bp in size. The *ACTB* was used as an internal control. All experiments were repeated three times. The primer sequences for other immuno-response genes and the amplified size for the RT-PCR are presented in Table 1.

### 2.7. Molecular Docking

The 3D structure files of the ligand CD (PubChem CID:6303), the protein NRP1(b1b2, the structure of b1b2 domains) (PDB ID:2QQI), and the RP1(b1, the structure of b1b2 domains) (PDB ID:1KEX) were obtained from the PubChem database (https://pubchem.ncbi.nlm.nih.gov/, accessed on 1 January 2023) [50] and the Protein Data Bank (PDB, https://www.rcsb.org/, accessed on 1 January 2023), respectively. Default docking studies were attempted to explore the binding mode of the suggested CD onto the 3D model of NRP1 using AUTODOCK tools 1.5.7 [51]. The crystal structure of the center of the NRP1 was placed as the center of the molecular docking box. The Vina algorithm was applied in this research. The maximum number of binding modes was nine. Default settings were used for all other parameters. Output files for the docking were saved as both ligand_out.pdbqt and log.txt files. The PyMol (v2.0) and the BIOVIA Discovery Studio Visualizer (v21.1.020298) were employed to visualize the binding interactions between CD and NRP1.

### 2.8. Cell Transfection and Syncytial Formation

Syncytia formations were regarded as hallmark cellular events for SARS-CoV-2 invasion [52]. The SARS-CoV-2 spike plasmid, carrying green fluorescent protein (GFP) fluorescence pCDH-CMV-HnCoV-S-EF1-copGFP, was purchased from Shanghai HedgehogBio Science and Technology Ltd. (Shanghai, China) The 293T-hACE2 cell lines were transfected via the SARS-CoV-2 spike plasmid for 24 h, then 20 μM of CD was added for another 24 h. The syncytial formation was examined using a ZOE Fluorescent Cell Imager (Bio-Rad Laboratories, Inc., Hercules, CA, USA). The NRP1 protein level was monitored using Western blotting.

### 2.9. Statistical Analysis

The statistical analysis was conducted using a *t*-test (two groups), expressed as a mean ± standard deviation (mean ± SD). The mean grayscale values and fluorescence area of individual fluorescence images were monitored using Image J software (Java 1.8.0_322). *p* < 0.05 was considered to be different, whereas *p* < 0.01 to be significantly different.

## 3. Results

### 3.1. Cordycepin (CD) Inhibits NRP1 Expression in Various Cells

**Figure 1:**
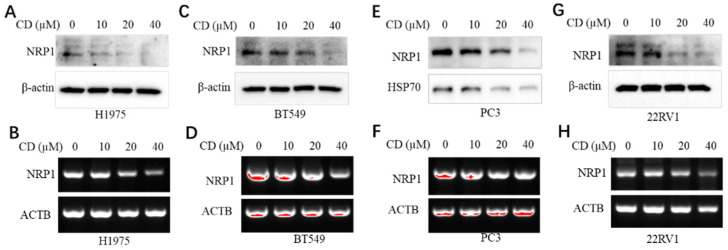
Cordycepin (CD) inhibits NRP1 expressions of both the mRNA and the protein in various cancer cell lines. (**A**,**B**) CD decreases NRP1 expressions in the H1975 lung cancer cells. (**C**,**D**) CD decreases NRP1 expressions in the BT549 breast cancer cells. (**E**,**F**) CD decreases NRP1 expressions in the PC3 prostate cancer cells. (**G**,**H**) CD decreases NRP1 expressions in the 22RV1 prostate cancer cells. (**A**,**C**,**E**,**G**) are for the NRP1 protein level of NRP1, and (**B**,**D**,**F**,**H**) are for the NRP1 mRNA level.

Some natural active components or small molecules could modulate gene expression. Cordycepin (CD) is an active gradient or natural product from traditional Chinese medicine. As an adenosine derivative, CD processes a diverse, broad spectrum of biological/pharmacological activities, such as anti-cancer, antimetastatic, anti-viral, antiprotozoal, antimalarial, antimicrobial, insecticidal, anti-inflammatory, antioxidant, and immunomodulatory/immunoregulatory [39]. Thus, the CD was used to test the impact on NRP1 expressions in human cancer cell lines. The results are shown in Figure 1, where CD inhibits the NRP1 expressions in both the protein and the mRNA in dosage-dependent manners in the H1975 cells (Figure 1A,B), the BT549 cancer cells (Figure 1C,D), the PC3 prostate cancer cells (Figure 1E,F), and the 22RV1 prostate cancer cells (Figure 1G,H), respectively.

### 3.2. Docking and Molecular Interaction Study of CD with NRP1

**Figure 2:**
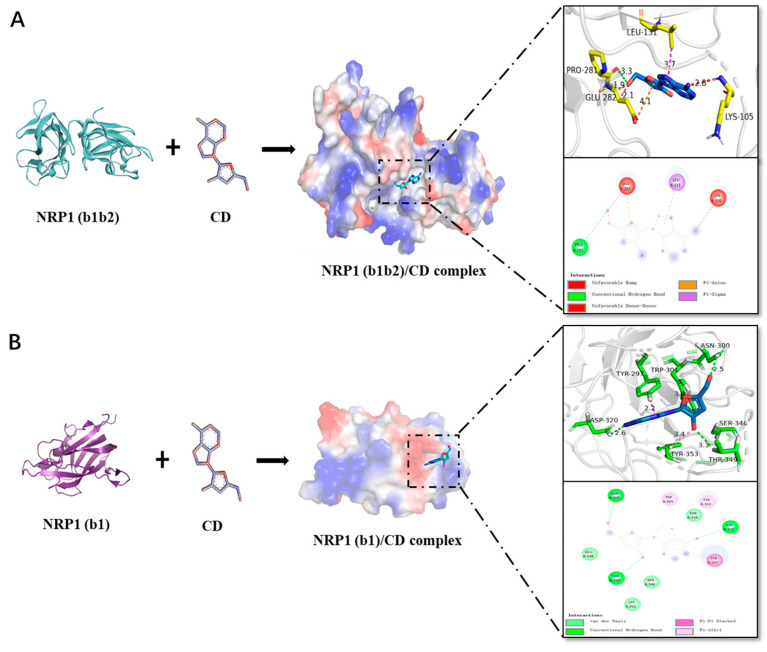
Binding mode of Cordycepin (CD) and NRP1 and two-dimensional illustration of interactions with NRP1 residues. (**A**) The left panel shows the three-dimensional structure for CD and NRP1(b1b2), respectively, the middle panel shows the three-dimensional conformational alignment for CD in the binding pocket, and the right panels show the binding mode of CD and the b1b2 domains of NRP1 (**upper**) and two-dimensional illustration of interactions with NRP1 b1b2 residues (**bottom**). (**B**) The left panel shows the three-dimensional structure for CD and NRP1 (b1), the middle panel shows the three-dimensional conformational alignment of CD in the binding pocket, and the right panels show the binding mode of CD and the b1 domains of NRP1 (**upper**) and two-dimensional illustration of interactions with NRP1 b1 residues (**bottom**). The image has been generated using PyMOL (v2.0) and BIOVIA Discovery Studio Visualizer software (v21.1.020298). In the binding pattern diagram, the red, orange, purple, pink, light pink, green and light green dashed lines represent unfavourable bump, unfavourable donor-donor, pi-sigma, pi-pi stacked, pi-alkyl, conventional hydrogen bond, van der waals and other interactions, respectively. Key binding residues of CD to NRP1(b1) and NRP1(b1b2) are indicated by yellow and green bars, respectively..

In silico analysis found that interfering with SARS-CoV-2 binds to NRP1 via small molecules seem to be potential candidates as novel anti-viral agents [31,32,33,34]. Folic acid, allleucovorin, and alimemazine may have the potential to prevent SARS-CoV-2 internalization by interacting with the S-protein/NRP1 complex [35,36]. Recently, Skrbic et al. used NRP1(b1b2), the structure of the b1b2 domains of NRP1, to perform a molecular docking study [35]. To this end, we also used NRP1(b1b2) for CD docking in silico, and the results are shown in Figure 2A and Table 2. The molecular docking results showed that the highest binding affinity between CD and NRP1 (b1b2) was −6.3 kcal/mol. CD can form a strong hydrogen bond with the residue of NRP1 (b1b2) Pro281 with a distance of 3.3 Å (Figure 2A, right upper). In addition, the five-membered aromatic ring of CD can form significant hydrophobic interactions with the residue Glu282 of NRP1 (b1b2) (Figure 2A). Through two-dimensional (2D) modeling, we observed that CD engages with NRP1(b1b2) via a variety of non-covalent bonds and interactions (Figure 2A, right bottom).

Recent studies have shown that the CendR sequence of the SARS-CoV-2 S-protein can bind to the NRP1 protein b1 domain to enhance virus entry into host cells [7,8]. However, when we used docking software (AUTODOCK tools 1.5.7) to analyze the interaction between the b1b2 domain of NRP1 and CD, we found that the position of CD docking was not located at the natural active site of the b1 domain as they reported. In order to further investigate this, we decided to dock the CD and the NRP1 b1 domain. The highest binding affinity of CD to NRP1 b1 was −6.5 kcal/mol (Table 2). Three hydrogen bonds of Asp 320, Asn 300, and Thr349 residues stabilized the target protein-binding molecule CD with distances of 2.6 Å, 2.5 Å, and 3.3 Å, respectively (Figure 2B, right upper). The benzene ring of CD has a Pi-Pi stacking with residue Tyr297 (Figure 2B, right upper). Further 2D modeling revealed diverse non-covalent bonds and interactions between CD and NRP1 (b1) (Figure 2B, right bottom).

### 3.3. CD Inhibits Syncytial Formation Likely through NRP1

**Figure 3:**
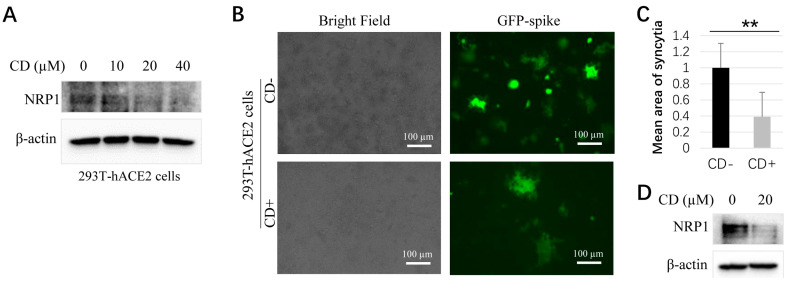
Cordycepin (CD) inhibits NRP1 expressions and syncytial formation in 293T-hACE2 cells. (**A**) NRP1 protein expressions in 293T-hACE2 cells with different amounts of CD treatment. (**B**) Representative images for syncytia formation in control without CD (CD−) and treated with CD (CD+) of 293T-hACE2 cells. (**C**) The quantitative results for (**B**). (**D**) NRP1 protein expression in 293T-hACE2 cells with CD treatment for (**B**). An unpaired student test was used for statistical analysis. “**”, *p* < 0.01.

A pathological hallmark for SARS-CoV-2 entry forms syncytia with multinucleated cells, evidenced in patients with COVID-19 [53]. Syncytium formation is required to participate in the S-protein of SARS-CoV-2 when host cells have the human *ACE2* gene [52]. First, we used 293T-hACE2 cells to treat with CD and found that CD decreased NRP1 protein expression in a dosage-dependent context (Figure 3A). After the CD treatment and transfection of SARS-CoV-2 spike plasmids with GFP fluorescence in 293T-hACE2 cells, the area of fluorescence of GFP-positive syncytia (Figure 3A, bottom panel) was significantly decreased when compared with the control cells, which indicate SARS-CoV-2 cell entry (Figure 3A, upper panel). The quantitative results are shown in Figure 3C. To investigate whether treatment with CD inhibited syncytia formation, at least partially, via NRP1, further Western blotting was performed, and the results in Figure 3D show that the level of the NRP1 protein is significantly downregulated in 293T-hACE2 cells treated with CD compared with control cells (Figure 3D). Therefore, the CD might inhibit the formation of syncytia via NRP1.

### 3.4. CD Roles in Immune Molecules and NRP1 Expression Analysis on Correlated Genes

**Figure 4:**
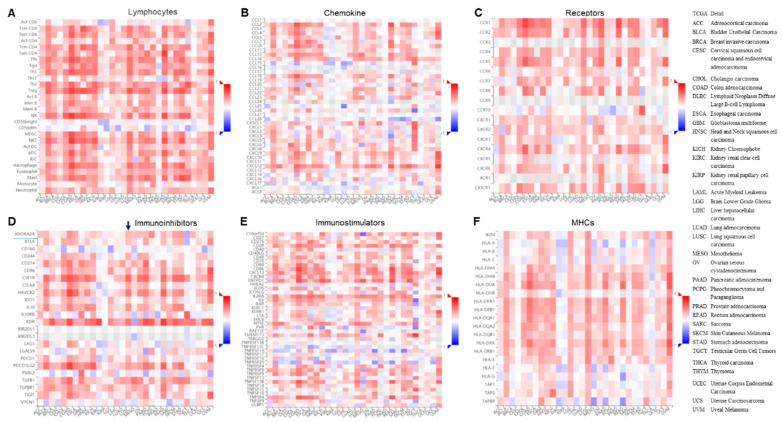
Associations of NRP1 expressions with a tumor–immune response in different cancers. (**A**) The associations between NRP1 expressions and lymphocyte response in cancers. (**B**) The associations between NRP1 expressions and chemokine response in cancers. (**C**) The associations between NRP1 expressions and receptor response in cancers. (**D**) The associations between NRP1 expressions and immunoinhibitor response in cancers. (**E**) The associations between NRP1 expressions and immunostimulator response in cancers. (**F**) The associations between NRP1 expressions and MHC response in cancers. Spearman associations between expressions of NRP1 and TILs (Y-axis) across human cancers (X-axis). The full names of cancer types are shown on the right.

We want to know the associations between the abundance of tumor-infiltrating lymphocytes (TILs) and expressions of NRP1 across human cancers, so an integrated repository portal for TISIDB was applied for the analysis. The results are shown in Figure 4, where we, interestingly, found significant associations between *NRP1* expressions and tumor–immune response in immune lymphocytes (Figure 4A), chemokines (Figure 4B), receptors (Figure 4C), immune inhibitors (Figure 4D), immunostimulators (Figure 4E), and major histocompatibility complex (MHC) molecules (Figure 4F) in most pan-cancers. Specifically, many immuno-response genes have been shown to be significantly changed, such as *CD28*, *CXCL12*, *CSF1R*, *KDR*, *CCR1*, *IL2RA*, etc. (Table 2).

**Figure 5:**
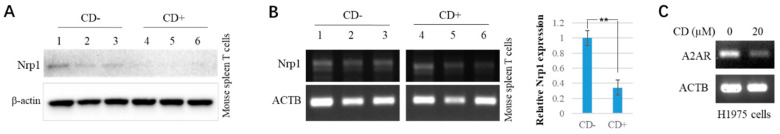
Cordycepin (CD) inhibits NRP1 expressions in lymphocytes in mice and cells. (**A**) CD inhibits NRP1 protein expressions in lymphocytes in vivo. (**B**) CD inhibits NRP1 mRNA expressions in lymphocytes in vivo. Quantitative data are shown in the right panel. **, *p* < 0.01. (**C**) CD inhibits A2AR mRNA expressions in the lung cancer cell line H1975.

Cancer cell lines treated with CD, an adenosine derivative, downregulated the NRP1 expression. The involvement of immune molecules from the adenosine/A2AR pathway has been described [54,55,56,57]. Our previous study showed that both adenosine derivatives, N6, N6-dimethyladenosine, CD, adenosine-inhibited DPP4/CD26 expression in cancer cells, and adenosine further revealed that it significantly suppresses the expression of the lymphocyte activating factor 3 (Lag3) in mice with AD injection. Thus, we wanted to know which immune molecules are influenced by CD and are correlated with a NRP1 downregulated expression. In mice treated with or without CD, isolated lymphocytes, Western blotting, and a semi-quantitative RT-PCR were performed to monitor the NRP1 expression. The results are shown in Figure 5, which shows that both protein (Figure 5A) and mRNA (Figure 5B) levels of NRP1 are significantly downregulated. Then, we further examined whether the above immuno-response genes were changed in those isolated lymphocytes from mice, and the results shown in Supplementary Figure S1 explain that the expressions for *Cd28*, *Cxcl12*, *Csf1r*, *Kdr*, *Ccr1*, and *Il2ra* are not significantly downregulated. These data implied NRP1 roles and mechanisms in both anti-cancers and anti-SARS-CoV-2 entry, probably through immuno-response genes/pathways, other than Cd28, Cxcl12, Csf1r, Kdr, Ccr1, and Il2ra in mice.

Then, we carefully looked into adenosine-mediated genes and found that the *A2AR* (*ADORA2A*) gene is relatively highly upregulated in lung cancer (lung squamous cell carcinoma, LUSC) (Figure 4D, arrow). Thus, we treated an adenosine derivative, CD, as the above-tested lung cancer cell line H1975, and a RT-PCR found that CD significantly inhibited *A2AR* expression (Figure 5C).

## 4. Discussion

NRP1 can act as a co-receptor to fascinate SARS-CoV-2 invasion into host cells [7,8,9,19]. Thus, it is important to investigate NRP1 expression regulation via small molecules and the potential role of SARS-CoV-2-infected cancer patients. In current studies, we revealed that adenosine derivatives CD-suppressed NRP1 expression in cancer cells, implying the therapeutic potential for anti-cancer. Our previous study showed that CD has been reported to inhibit tumorigenesis and cancer metastasis/invasion both in vitro and in vivo [40,41].

Cordycepin (CD) is one of the main active gradients that was isolated from traditional Chinese medicine mushrooms *Ophiocordycep*s Sinensis (Berk.) [58] and *Cordyceps militaris* Link [59]. As a well-known natural adenosine analog of fungal origin, the CD can be synthesized, making it more possible for the study. CD processes a diverse, broad spectrum of biological/pharmacological activities, such as anti-cancer, antimetastatic, anti-viral, antiprotozoal, antimalarial, antimicrobial, insecticidal, anti-inflammatory, antioxidant, immunomodulatory/immunoregulatory, antileukemic, antiproliferative, apoptosis inducer, antifibrotic, antihyperglycemic/antidiabetic, antihyperlipidemic, antitachycardic, antihypercholesterolemic, antiarrhythmic, angiogenic, antihypertensive, antithrombotic/fibrinolytic/thrombolytic, anti-ischemic, reperfusion therapy, antistroke, hepatoprotective, renal functions improver/nephroprotective, chondrogenesis promoter, antiarthritic, antiosteoporotic, intervertebral disc regenerator, cystic fibrosis, acute lung inflammation/injury healer, chronic obstructive pulmonary disease (COPD), antihypoxic, cough/common cold suppressant, antidepressant, natural endurance booster, antifatigue, pain killer/analgesic, erythropoiesis stimulator, antiparkinsonian, neuronal regenerator, antisleep disorders, antiaging, aphrodisiac, sexual enhancer, spermatogenic, antiinfertility, some toxins antidote, and cosmeceutical [40,41,60,61,62]. These aforementioned activities make CD one of the most promising drugs with pharmacological and therapeutic potential [63,64,65]. Importantly, CD has recently discovered potent inhibitory activities on SARS-CoV-2 replication [60,66] that could contribute to treatments for COVID-19 [67]. This probable inhibitory affinity for CD against the principal protein targets of SARS-CoV-2 included the S-protein, enzymes of protease (Mpro), and the RNA-dependent RNA polymerase (RdRp) [62]. Our study found that CD inhibits the NRP1 expression of both protein and mRNA levels in a dosage-dependent manner in various cancer cell lines, implying the therapeutic potential for anti-SARS-CoV-2 and anti-cancers. This is the first study to identify that CD can inhibit NRP1 expression. Recently, we also showed that CD inhibited the expressions for CTSL, CD147, DPP4/CD26, furin, and SARS-CoV-2 entry proteins [38,48,68]. Moreover, CD might inhibit the formation of syncytia via NRP1, at least in part. Molecular docking results showed that there were multiple hydrogen bonds and hydrophobic interactions between CD and NRP1, with the highest binding affinity of CD to NRP1 b1 being −6.5 kcal/mol. Strikingly, CD binding exhibited strong similarity to the structure of the NRP1 b1 domain in the complex of the S1 Cend R peptide-NRP1 b1 and the known NRP1 inhibitors-NRP1 b1 [8,69]. This shows some common interactions, including the binding of the 320th aspartic acid (Asp) and the 300th asparagine (Asn) to the NRP1 b1 domain, which may be through different mechanisms, not interfering with NRP1-mediated SARS-CoV-2 S-protein initiation rather than inhibiting NRP1 expression via CD, thereby inhibiting the virus’s subcellular entry.

Recently, Hou et al. [70] investigated interactions between NRP1 and the S-protein of SARS-CoV-2 under a custom-built atomic force microscopy (AFM) and found biophysical characteristics of interactions with various S-protein fragments, including the S-protein trimer and the receptor-binding domain (RBD). Predicting via AlphaFold2 and MD simulation in NRP1 a1a2b1b2 domains between residue 22 and 591 and the SARS-CoV-2 S-protein RBD between residue 319 and 537 revealed two models (models 2 and 3). Model 2 showed that the receptor-binding motif (RBM) was in close contact with the NRP1 b1 domain between residues 254 and 403. The representative interacting residue pairs of RBM (a crucial binding sequence of RBD) and NRP1 were K458-D320, F456-Y297, N501-K351, and T500-K350 (RBM-NRP1). However, in model 3, the interacting residues are slightly different from model 2, where the Q493 and Y505 (RBD-NRP1) of the RBD were involved in the interface interactions, Q493 showed a charge–charge interaction with K351 and Y297, and Y505 showed hydrophobic interactions with Y297. The residue Y297 of NRP1 also interacted with the N501 of the RBD, and F456 had close contact with the residue W411 of NRP1 via π–π interactions. The RBM-NRP1 interface is similar to those in the RBD-ACE2 interface. Nevertheless, based on the interaction overlapping for NRP1 b1 between CD and the S-protein of SARS-CoV-2, CD not only inhibits NRP1 expression but also interrupts the interaction of the SARS-CoV-2 S-protein, thereby inhibiting the virus’s subcellular entry.

NRP1 expression is upregulated in many cancer types, including kidney renal clear cell carcinoma (KIRC) and kidney renal papillary cell carcinoma (KIRP), compared to the matched healthy tissues and is positively correlated with the survivability rate of KIRC patients [71]. The high-affinity binding between the SARS-CoV-2 S-protein and NRP1 suggested that this binding might play a role in COVID-19 severity since their binding promotes SARS-CoV-2 invasion through NRP1. It is well-known that NRP1 is expressed in normal kidney tubule tissue, KIRC, and KIRP, implying a possible target for budding therapeutics in cancers [72]. NRP1 is also highly expressed in neuronal cells and olfactory epithelium, thus contributing to the SARS-CoV-2 entering the central nervous system, causing a pathological complication, and enhancing the deterioration of glioblastoma or brain tumors [73].

Additionally, we interestingly reveal significant associations between *NRP1* expressions and the tumor–immune response in immune lymphocytes, chemokines, receptors, immunostimulators, immune inhibitors, and MHC molecules in almost all pan cancers. Specifically, many immuno-response genes are significantly changed, such as *CD28*, *CXCL12*, *CSF1R*, *KDR*, *CCR1*, *IL2RA*, etc. Thus, we further examined whether the above immuno-response genes were changed in those isolated lymphocytes from mice and found that the expressions for *Cd28*, *Cxcl12*, *Csf1r*, *Kdr*, *Ccr1*, and *Il2ra* are not significantly downregulated, implying NRP1 roles and mechanisms in both anti-cancers and anti-SARS-CoV-2 entry, probably through immuno-response genes/pathways, other than *CD28*, *CXCL12*, *CSF1R*, *KDR*, *CCR1*, and *IL2RA*.

Of course, we should note that we have analyzed those genes in mice, not humans; there may be differences between different species when treated with CD. With this regard, we carefully looked into adenosine-mediated genes and found that the *A2AR* gene is relatively highly upregulated in lung cancer. The A2AR is a novel immune checkpoint gene, and Fong et al. reported that A2AR antagonists can be used for immunotherapy for patients with refractory renal cell cancer [55]. Thus, we treated an adenosine derivative, CD, in the lung cancer cell line H1975 and found that CD significantly inhibited A2AR expression, highlighting the CD/A2AR/immunotherapy pathway in anti-cancer and anti-SARS-CoV-2.

Moreover, NRP1 also facilitated other viral infections, such as the (EBV) [26], the PRV [27], the mCMV [28], the HTLV-1 and HTLV-2 [29], and CD inhibited NRP1 expression and viral syncytial formation, highlighting therapeutic significances for anti-different viruses.

## 5. Conclusions

In conclusion, our study highlights the significance of NRP1 expression regulation, and the natural product CD downregulated the expressions not only in NRP1 but also in immune molecules such as A2AR, implying a NRP1 mechanism probably through immuno-response pathways, providing a potential CD therapy for anti-cancer and anti-viral diseases, including COVID-19.

## Figures and Tables

**Table 1:** Immuno-response genes, primer sequences, and amplified size for RT-PCR in mice.

Gene Name	Primers	Sequence (from 5′-3′)	GenBank No.	Size (bp)
Cd28	RT-mCD28-L	acaacgagaggagcaatgga	NM_007642.4	401
	RT-mCD28-R	gcccagtagaggtccaaagt		
Cxcl12	RT-mCXCL12-L	ctttcactctcggtccacct	NM_001012477.2	258
	RT-mCXCL12-R	gcaacaatctgaagggcaca		
Csf1r	RT-mCSF1R-L	gcctcttcctctgttccctt	NM_001037859.2	372
	RT-mCSF1R-R	attcagggtccaaggtccag		
Kdr	RT-mKDR-L	ggagtctgtgcctgagaact	NM_010612.3	440
	RT-mKDR-R	acagaggcgatgaatggtga		
Ccr1	RT-mCCR1-L	ttggaaccagagagaagccg	NM_001295.3	259
	RT-mCCR1-R	agaaatggccaggttcagga		
Il2ra	RT-mIL2RA-L	acaagaacggcaccatccta	NM_008367.3	525
	RT-mIL2RA-R	agtctgtggtggttatgggg		

**Table 2:** The binding affinity of the CD-NRP1 complex and contacting residues of NRP1.

Protein−Ligand Complex	Binding Affinity (kcal/mol)	NRP-1-Contacting Residues
CD-NRP1(b1b2)	−6.3 #	Lys105, Leu131, Pro281 *, Glu282
CD-NRP1(b1)	−6.5 #	Tyr297, Asn300 *, Trp301, Asp320 *, Thr349 *, Tyr353

# the highest biding affinity; * hydrogen bond interaction sites.

## Data Availability

Data are contained within the article.

## Supplementary Materials

The following supporting information can be downloaded at: https://www.mdpi.com/article/10.3390/microorganisms11122953/s1, Figure S1: The mRNA expressions of *Cd28*, *Cxcl12*, *Csf1r*, *Kdr*, *Ccr1*, and *Il2ra* when treated with CD in mice.
