# Supplementary material for: Natural Product Cordycepin (CD) Inhibition for NRP1/CD304 Expression and Possibly SARS-CoV-2 Susceptibility Prevention on Cancers
Source: Microorganisms. 2023 Dec 10;11(12):2953. doi: 10.3390/microorganisms11122953 (PMC10745444; doi:10.3390/microorganisms11122953)
Supplement: Supplementary file 1 [file microorganisms-11-02953-s001.zip › microorganisms-2744701-supplementary.pdf]

Supplementary figures

Supplementary figure

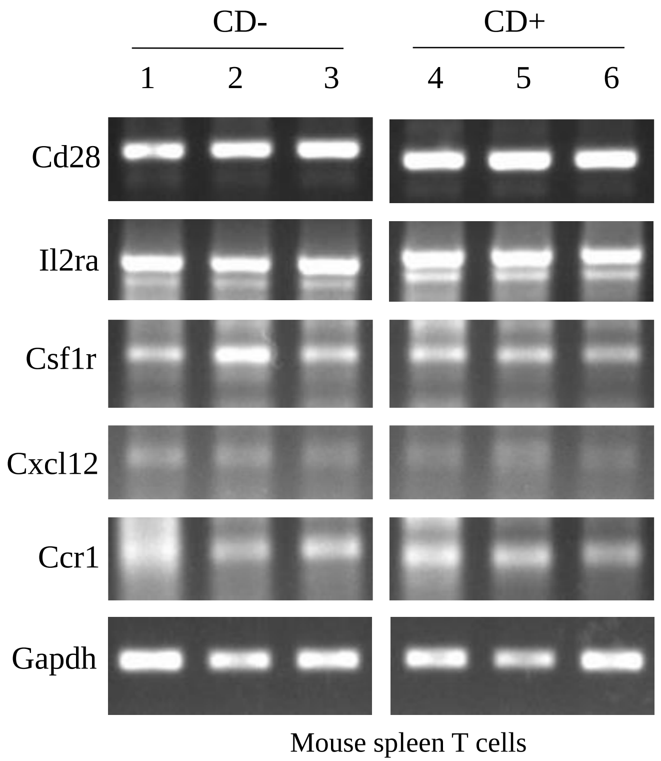

Supplementary figure S1. The mRNA expressions of *Cd28*, *Cxcl12*, *Csf1r*, *Kdr*, *Ccr1*, and *Il2ra* when treated with CD in mice.
